# Three-dimensional characterization of mandibular asymmetry in craniofacial microsomia

**DOI:** 10.1007/s00784-020-03302-8

**Published:** 2020-05-07

**Authors:** Yun-Fang Chen, Frank Baan, Robin Bruggink, Ewald Bronkhorst, Yu-Fang Liao, Edwin Ongkosuwito

**Affiliations:** 1 Department of Craniofacial Orthodontics, Chang Gung Memorial Hospital, Taipei, Taiwan; 2 Graduate Institute of Dental and Craniofacial Science, Chang Gung University, Taoyuan, Taiwan; 3 Department of Dentistry, Section of Orthodontics and Craniofacial Biology, Radboud University Medical Center, Philips van Leydenlaan 25, 6525 EX Nijmegen, The Netherlands; 4 Radboudumc 3DLab, Radboud University Medical Center, Nijmegen, The Netherlands; 5 Department of Dentistry, Section of Preventive and Restorative Dentistry, Radboud University Medical Centre, Nijmegen, The Netherlands; 6 Department of Craniofacial Orthodontics, Chang Gung Memorial Hospital, Taoyuan, Taiwan; 7 Amalia Cleft and Craniofacial Centre, Radboud University Medical Centre, Nijmegen, The Netherlands

**Keywords:** Craniofacial microsomia, Hemifacial microsomia, Mandibular asymmetry, Facial asymmetry, Cone-beam computed tomography

## Abstract

**Objectives:**

This study aimed to investigate the three-dimensional (3D) mandibular asymmetry in craniofacial microsomia (CFM) and its association with the Pruzansky–Kaban classification system.

**Materials and methods:**

Cone-beam computed tomography images of 48 adult CFM cases were collected. The asymmetry of the mandibular body and ramus was analyzed with 3D landmarks. The mirrored mandibular model was registered on the original model, yielding a color-coded distance map and an average distance (i.e., asymmetry score) to quantify the overall mandibular asymmetry.

**Results:**

The lengths of the mandibular body and ramus were significantly shorter on the affected than the contralateral side (*p* < 0.001). The ANB (*p* = 0.009), body and ramal lengths (both *p* < 0.001), and body and ramal length asymmetry (both *p* < 0.05) were significantly different between mild (types I/IIA) and severe (types IIB/III) cases. The mandibular asymmetry score correlated with mandibular body length asymmetry (*r* = 0.296, *p* = 0.046). CFM mandibles showed high variability in shape asymmetry.

**Conclusions:**

CFM patients showed distinct body and ramal length asymmetries. In severe cases, mandibles were smaller, more retruded, and more asymmetric in length. The mandibular shape asymmetry was highly variable regardless of the Pruzansky–Kaban types, being a determinant in the extent of overall mandibular asymmetry.

**Clinical relevance:**

The 3D morphologic analysis provides better insights into real mandibular asymmetry. Although the Pruzansky–Kaban classification was applied, high individual variability of the mandibular morphology still existed within the types. Therefore, individualized analyses and treatment plans for CFM patients are highly recommended.

## Introduction

Craniofacial microsomia (CFM) is the third most common congenital craniofacial anomaly after cleft lip and palate and craniosynostosis, with an incidence ranging from 1:3500 to 1:5600 in live births [1, 2]. In CFM, embryonic development of the nasal placode and first and second pharyngeal arches is disturbed [3, 4], but the etiology is not fully clarified. The most plausible pathogenic models of CFM are vascular abnormality and hemorrhage or neurocristopathy [5, 6]. Patients with CFM are characterized by hypoplasia of the mandible (89–100% of cases) and ear (66–99% of cases), primarily on one side, producing the associated facial asymmetry [7–9]. Bilateral involvement occurs in 5–15% of CFM patients, and mandibular asymmetry remains a typical feature for them [4, 7, 8].

Mandibular asymmetry in CFM results from unilaterally dominant hypoplasia in the skeletal or soft tissue structures, in addition to having functional or neuromuscular origins [10–12]. Some reports suggest that mandibular growth in CFM is constant and that asymmetry does not increase over time [13–17], although this claim remains controversial. For moderate to severe mandibular asymmetry especially, management of soft tissue asymmetry often cannot be addressed efficiently, and the esthetic outcome will not be satisfactory before the skeletal frame is restored. Various treatments, including autogenous grafting, distraction osteogenesis, orthognathic osteotomy, and prosthetic replacement [18, 19], have been proposed and focus mainly on improving the skeletal mandibular asymmetry. Nevertheless, no consensus exists on treatment protocols regarding technique, sequence, or timing because of lack of agreement about asymmetric growth and high phenotypical heterogeneity of the mandibular deformity. In the meantime, the Pruzansky–Kaban classification system, which is based on the severity of temporomandibular joint and mandibular deformity, is the most commonly used tool in planning interventions [20, 21]. Although this system was developed based on two-dimensional (2D) radiography, it can be applied in modern three-dimensional (3D) images (i.e., computed tomography [CT] or cone-beam CT [CBCT]) [4]. In addition to a schematic description of the mandibular deformity that this classification system provides, understanding the etiology, growth patterns, and 3D morphology of the mandibular malformation is necessary for an optimal treatment plan for the asymmetry. Most described analyses have involved 2D images (i.e., cephalograms, orthopantomograms, or photographs) [14, 16, 17, 22, 23]. The outcome of the 2D studies, however, has been inconclusive because of overlapping structures, magnification variability, or image distortions that can lead to misinterpretations [24].

CBCT or CT has been proposed as the better tool for facilitating access to all target structures, accurate measurements, and analyses in three dimensions (e.g., linear, angular, and volumetric measurements; topographical analysis; 3D superimpositions). However, previous CBCT and CT studies analyzing mandibular asymmetry in CFM have presented results offering limited information (i.e., merely the length of ramus, condyle, or corpus; or the volume of condyle) [11, 12, 24–27]. Solem et al. published a more comprehensive elucidation of mandibular asymmetry in CFM [28], describing asymmetry location and direction through comparison of the 3D CBCT models with their mirrors, but their sample had only 9 patients. Most 2D and 3D studies on the CFM mandibular asymmetry have focused on pediatric patients, and information for adults is scarce.

The aim of this CBCT study therefore was to evaluate mandibular asymmetry in adult patients with CFM. Furthermore, the association of mandibular asymmetry and its characteristics with the severity of the mandibular deformity based on the Pruzansky–Kaban classification system was investigated.

## Materials and methods

### Patients

This study included 48 Taiwanese adults (age > 16 years) with CFM who were consecutively selected at the Chang Gung Craniofacial Center between 2009 and 2018, based on the following criteria: (1) no congenital craniofacial syndromes other than CFM or Goldenhar syndrome, (2) no history of craniofacial surgery or trauma, and (3) available CBCT before orthodontic treatment or orthognathic surgery.

### CFM diagnosis

The diagnosis of CFM was based on clinical signs and symptoms and a review of the CBCT of the craniofacial skeleton. According to the Pruzansky–Kaban classification system [29, 30], two orthodontists further divided the CFM patients into two groups (mild: types I and IIA; severe: types IIB and III), reaching consensus in cases of initial disagreement after discussion. The presence of Goldenhar syndrome was screened for based on the triad of CFM, ocular dermoid cysts, and spinal anomalies and can be considered a CFM variant that is present in about 10% of cases [31].

### CBCT

CBCT of the head and neck was taken using an i-CAT 3D Dental Imaging System (Imaging Sciences International, Hatfield, PA, USA) with the following parameters: 120 kVp, 0.4 mm voxel size, 40 s scan time, and 16 cm × 16 cm field of view. All patients were scanned with the head in a natural position. Throughout the scan, patients were asked to bite in maximum intercuspidation, relax their lips, and not swallow.

Images were stored in the Digital Imaging and Communications in Medicine (DICOM) format. Maxilim (Medicim NV, Mechelen, Belgium) was used for 3D volumetric rendering of the head. To evaluate the sagittal skeletal relationship, a plane connecting the sella, nasion, and A-point was created for each head model, and the angulations of SNA, SNB, and ANB were measured on the same plane (i.e., SNA plane) to obviate incorporation of the transverse discrepancy of nasion, A-point, and B-point [32]. The mandible of each patient then was manually isolated from the head model for the following analyses.

**Fig. 1:**
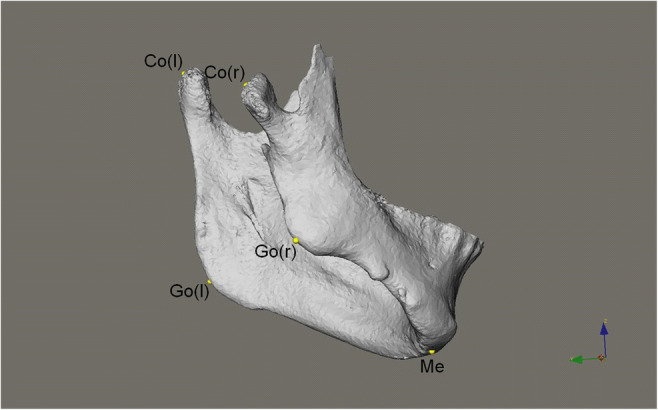
Landmarks for linear measurements of mandible: Me, menton; Go(l), gonion left; Go(r), gonion right; Co(l), condylion left; Co(r), condylion right

Five landmarks (i.e., menton and the bilateral gonia and condylions) [33] were designated on each mandibular model for the length measurements of the mandibular body and ramus (Table 1 and Fig. 1). These five landmarks were not applied to type III deformity mandibles because the structures of interest were partly or almost missing. Multi­planar reconstruction views were used to identify the landmarks when necessary. In addition to the absolute values of the length differences between the bilateral mandibular body and ramus, ratios of the shorter to the longer lengths of the mandibular body and ramus were calculated. A ratio close to 1 indicated symmetry between bilateral sides.

**Table 1:** Landmarks and linear measurements used for the mandibles

	Symbol	Definition
Landmarks		
Menton	Me	The most inferior midpoint of the chin on the outline of the mandibular symphysis
Gonion	Go	The point at each mandibular angle that is defined by dropping a perpendicular from the intersection point of the tangent lines to the posterior margin of the mandibular vertical ramus and inferior margin of the mandibular body or horizontal ramus
Condylion	Co	The most postero-superior point of each mandibular condyle
Linear measurements		
Mandibular body length	Go-Me	The distance between Go and Me
Mandibular ramus length	Co-Go	The distance between Co and Go

**Fig. 2:**
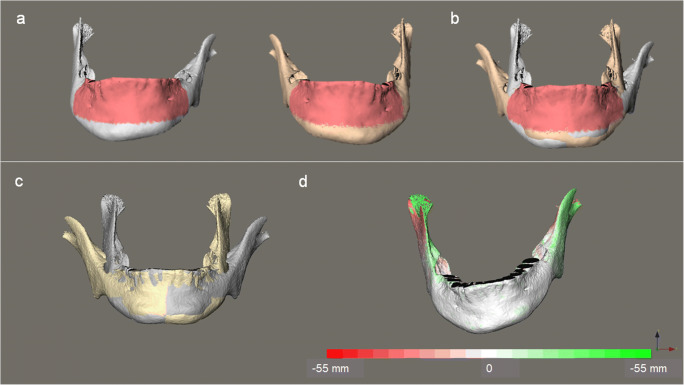
Steps of analysis of mandibular asymmetry. **a** The mandibular model (gray) was imported into MED software, and a mirrored model of mandible (yellow) was created. Pre-defined registration regions were selected (pink) on both models. **b** The mirrored model was registered on the original model at the pre-defined registration region based on the iterative closest point algorithm. **c** The registered pair of models was imported into Maxilim software. **d** The inter-surface distances between the paired models were calculated and visualized as a color-coded distance map

Overall asymmetry of the 3D mandibular model was analyzed by using a mirroring technique. In an in-house created software, MED, which is based on Open Inventor® (version 9.9.10, Bordeaux, France), a mirrored model of the mandible was created along an arbitrary plane and manually approximated toward the original model. The region for the final automated surface-based registration of the two models was confined to the labial and lingual surfaces of the mandibular body mesial to bilateral second molars. The lower boundary of the registration region was further defined by a plane. This plane was passing through the highest point of the lower border of the mandibular body between bilateral second molars, and parallel to a second plane that connected the infradentale (i.e., the highest anterior point of alveolar borer between the mandibular central incisors) and the highest buccal points of alveolar border between the first and second molars on each side of the mandible. As a result, the vertical dimension of the registration region was generally consistent and symmetric between bilateral sides. The registration was based on the iterative closest point algorithm. The registered pair of models was imported into Maxilim software, and the average value of the absolute inter-surface distances between the two models was computed yielding an asymmetry score to quantify the asymmetry between the left and right sides of the original mandibular model. Additionally, the location, magnitude, and directionality of the mandibular asymmetry were illustrated in a color-coded distance map (Fig. 2).

### Reliability

To assess intra-examiner reliability, the CBCT segmentation and measurements of 10 randomly chosen patients were conducted by one investigator, which was repeated 1 month after the initial session. To assess inter-examiner reliability, a second investigator independently conducted the same process for the same CBCT images. The intra- and inter-examiner reliabilities were tested using Pearson correlation coefficients. A paired *t*-test was used to evaluate systematic differences between the CBCT measurements. The random error in measurements was calculated with the duplicate measurement error, which was calculated by dividing the standard deviation (SD) by √2. For all tests, the significance level was set at *p* < 0.05.

### Statistical analysis

The Statistical Package for Social Sciences for Windows 24 (SPSS 24, IBM Corp., NY, USA) was used for statistical analysis. All descriptive statistics were presented as mean ± SD. Patient characteristics were compared between groups using independent *t*- or Fisher’s exact test when indicated. A paired *t*-test was used to compare the differences of CBCT measurements between the affected and contralateral sides of CFM and between the body and ramus. To compare the difference in CBCT measurements between different groups, an independent *t*-test was used. The correlations between CBCT measurements and patient characteristics (e.g., age, sex, severity of CFM) were assessed using Pearson or Spearman correlation analysis when indicated. All statistical tests were two-sided, with *p* < 0.05 considered statistically significant.

## Results

### Patient characteristics

The CFM deformities of the 48 recruited patients were all unilateral (30 women and 18 men; mean age, 20.0 ± 2.9 years; range, 16.4 to 31.4 years). A total of 36 patients were in the mild group (19 women and 17 men; mean age, 20.4 ± 3.0 years), and 12 were in the severe group (11 women and 1 man; mean age, 18.8 ± 2.2 years). No patient was diagnosed with Goldenhar syndrome (Table 2).

**Table 2:** Patient characteristics^a^

	CFM patients (*n* = 48)	Mild group^b^ (*n* = 36)	Severe group^b^ (*n* = 12)	*p* (mild vs. severe)
Age at CBCT (years (range))	20.0 ± 2.9 (16.4 to 31.4)	20.4 ± 3.0 (17.3 to 31.4)	18.8 ± 2.2 (16.4 to 24.8)	0.099^c^
Gender (*n*)				0.018^d^
Female	30	19	11	
Male	18	17	1	
Cephalometric analysis (degrees)				
SNA	78.53 ± 4.51	78.63 ± 4.43	78.23 ± 4.95	0.798^c^
SNB	74.91 ± 5.73	75.71 ± 5.33	72.51 ± 6.45	0.094^c^
ANB	5.25 ± 3.01	4.61 ± 2.51	7.18 ± 3.64	0.009^c^
CFM affected side (*n*)				0.726^d^
Right side	34	26	8	
Left side	14	10	4	
Bilateral sides	0	0	0	
Pruzansky–Kaban classification (*n*)				
Type I	22	22	–	
Type IIA	14	14	–	
Type IIB	10	–	10	
Type III	2	–	2	
Presence of Goldenhar syndrome (*n*)	0	0	0	

^a^Data are means ± SD except where otherwise indicated

^b^Patients were divided into mild and severe groups based on the Pruzansky–Kaban classification

^c^Independent *t*-test

^d^Fisher’s exact test

### Method reliability

For measurements of mandibular lengths and asymmetry scores, both the intra- and inter-examiner reliabilities were excellent (Pearson correlation coefficients ≥ 0.98). The paired *t*-test showed no significant difference in the measurements (Table 3).

**Table 3:** Results of the inter- and intra-observer reliability analyses

Parameters	*r*	DME	Mean difference	95% CI	*p*^a^
Intra-examiner variability					
Mandibular asymmetry score (mm)	0.997	0.17	− 0.03	− 0.21 to 0.14	0.665
Mandibular body length (mm)	0.993	1.30	− 0.55	− 1.86 to 0.76	0.368
Mandibular ramal length (mm)	0.989	0.89	0.24	− 0.66 to 1.14	0.561
Inter-examiner variability					
Mandibular asymmetry score (mm)	0.991	0.20	− 0.09	− 0.30 to 0.11	0.339
Mandibular body length (mm)	0.999	0.52	− 0.44	− 0.97 to 0.09	0.091
Mandibular ramal length (mm)	0.997	0.47	− 0.46	− 0.94 to 0.02	0.057

*r*, Pearson correlation coefficient; *DME*, duplicate measurement error; *CI*, confidence interval

^a^Paired *t*-test

### Mandibular characteristics

The ANB angle in the severe group was significantly larger than that in the mild group (*p* = 0.009) (Table 2). In the severe group, body lengths of the affected (*p* = 0.005) and contralateral sides (*p* = 0.003) and ramal lengths of the affected (*p* = 0.001) and contralateral sides (*p* = 0.005) were significantly shorter than those in the mild group (Table 4, columns).

**Table 4:** Mandibular body and ramal lengths in patients with CFM^a^

Patient group	Mandibular body length (Go-Me) (mm)					Mandibular ramal length (Co-Go) (mm)				
	Affected side		Contralateral side		*p* (affected vs. contralateral)	Affected side		Contralateral side		*p* (affected vs. contralateral)
	Mean	SD	Mean	SD		Mean	SD	Mean	SD	
CFM (*n* = 46)	74.57	9.02	83.16	5.73	< 0.001	41.62	9.76	58.60	6.38	< 0.001
Mild CFM (*n* = 36)	76.50	7.26	84.21	5.63	< 0.001	44.08	9.10	59.73	5.79	< 0.001
Severe CFM (*n* = 10)	67.63	11.57	79.37	4.50	0.002	32.79	6.59	54.51	7.05	< 0.001
*p* (mild vs. severe)	0.005		0.003			0.001		0.005		

*CFM*, craniofacial microsomia; *SD*, standard deviation

^a^The length measurements were not performed in the two CFM cases involving type III deformity

### Mandibular asymmetry in body and ramal lengths

The body and ramal lengths on the affected side were significantly shorter than those on the contralateral side within each group (i.e., total patients, and the mild and severe groups) (all *p* < 0.01) (Table 4, rows). The body (*r* = 0.695, *p* < 0.001) and ramal (*r* = 0.361, *p* = 0.014) lengths on the affected side were significantly positively correlated with those on the contralateral side for the 46 CFM patients (two patients with type III deformity were not included in this analysis). The absolute body length difference was significantly larger in the severe group than that in the mild group (*p* = 0.027). The body (*p* = 0.009) and ramal (*p* = 0.025) length ratios in the severe group also were significantly smaller than those in the mild group (Table 5, rows).

**Table 5:** Mandibular asymmetry in body and ramal lengths in patients with CFM^a^

Mandibular parameters	CFM (*n* = 46)		Mild CFM (*n* = 36)		Severe CFM (*n* = 10)		*p* (mild vs. severe)
	Mean	SD	Mean	SD	Mean	SD	
Absolute body length difference (mm)	8.90	6.07	7.87	5.47	12.62	6.93	0.027
Absolute ramal length difference (mm)	17.07	9.37	15.78	8.57	21.72	11.05	0.076
*p* (body vs. ramus)	< 0.001		< 0.001		0.067		
Body length ratio (%)	89.22	7.54	90.71	6.44	83.85	9.07	0.009
Ramal length ratio (%)	71.10	15.34	73.73	13.99	61.62	16.96	0.025
*p* (body vs. ramus)	< 0.001		< 0.001		0.006		

*SD*, standard deviation

^a^The length measurements were not performed in the two CFM cases involving type III deformity

The absolute length difference of the mandibular ramus was significantly greater than the mandibular body for the 46 CFM patients and the mild group (both *p* < 0.001). The length ratio of the mandibular ramus was significantly smaller than the mandibular body within each group (all *p* < 0.01) (Table 5, columns).

### Overall mandibular asymmetry

The mean asymmetry score of the mandible (i.e., the average value of the absolute inter-surface distances between the mirrored and original mandibular models) was 5.44 ± 2.40 mm for the 48 CFM patients. The difference in the mandibular asymmetry score was insignificant between the mild and severe groups (5.21 ± 1.93 mm for the mild group, 6.14 ± 3.48 mm for the severe group; *p* = 0.392). This score was significantly positively correlated with the absolute mandibular body length difference (*r* = 0.296, *p* = 0.046) (Table 6).

**Table 6:** Correlation between mandibular asymmetry and patient and mandibular characteristics

	Mandibular asymmetry score	
	Correlation coefficient^a^	*p*
Age	− 0.148	0.314
Gender	0.096^b^	0.515
CFM severity	0.094^b^	0.526
ANB	0.205	0.163
SNB	− 0.234	0.109
Absolute mandibular body length difference	0.296	0.046
Mandibular body length ratio	− 0.284	0.056
Absolute mandibular ramal length difference	0.027	0.860
Mandibular ramal length ratio	0.006	0.968

^a^Pearson correlation coefficient

^b^Spearman correlation coefficient

**Fig. 3:**
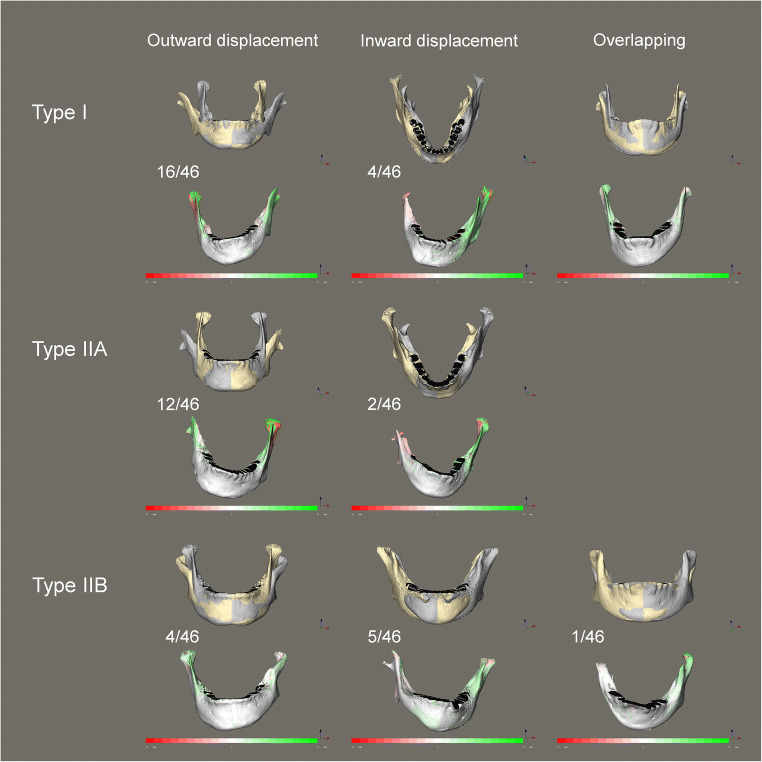
Cases demonstrating the high variability of mandibular shape asymmetry in CFM. The superimpositions of the original mandibular model (gray) and mirrored model (yellow) along with the color-coded distance maps showed that the affected ramus would be displaced outside or inside, or overlap the contralateral ramus. The prevalence of the ramal displacement in different directions among the 46 CFM patients (two cases with type III deformity lacked the ramus on the affected side and thus were not taken into calculation) was indicated next to the mandibular models. The color-coded scale was from − 55 to 55 mm

No specific trend in color pattern could be identified among the color-coded distance maps, indicating no direction preference for the deviation of the affected ramus in CFM: the affected posterior hemimandible might be displaced inward or outward relative to the mirrored contralateral posterior hemimandible. However, when focusing on the upper part of the affected ramus, in more than half of the cases (i.e., 32 of 46 cases with type I, IIA, and IIB deformities), displacement was outward relative to the mirrored contralateral ramus (Fig. 3).

## Discussion

The expression of the mandibular deformity in CFM patients is heterogeneous [11, 29, 34], and the mandibular characteristics differ from the normal population with regard to the size, shape, and sagittal and vertical discrepancies relative to the maxilla and cranial base [17, 28, 35, 36]. Therefore, instead of comparing the mandibular morphology of the CFM group with that of the general population, this study involved comparisons only among CFM patients (i.e., affected vs. contralateral side, mild vs. severe) to provide information on the mandibular asymmetry with a greater practical relevance for CFM treatment. The high SD values for length and asymmetry scores and varied color patterns of the distance maps identified here re-emphasize the morphological diversity of CFM mandibles.

To the authors’ knowledge, this study is the largest reported so far to rely on 3D mandibular asymmetry analyses in adult CFM patients. Two methods were applied. The first used 3D landmarks to analyze the linear dimensions of the mandible, which showed excellent intra- and inter-examiner reproducibilities. The results could be interpreted easily and applied to clinical practice, and the similar landmark-based analysis methods make possible a comparison with previous findings in still-growing CFM patients. Nevertheless, the 3D details of the mandibular asymmetry could not be captured without the inclusion of a large number of additional mandibular landmarks, which inevitably means incorporating landmarks with a lower degree of reproducibility and questionable improvement in the resulting information [35]. For this reason, a second method of mirroring and superimposition of 3D models was applied that facilitated 3D asymmetry analysis of the entire mandibular surface. The discrepancies in size and shape between the two sides of the mandible were calculated as distance values. Through generation of a color-coded distance map, both the amount and the location and direction of the asymmetry could be visualized. The numerous distance values were averaged so that the overall asymmetry of each mandible could be quantified as a single number (i.e., mandibular asymmetry score).

**Fig. 4:**
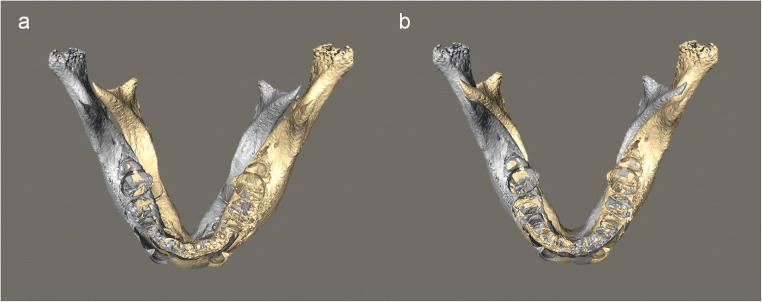
An example illustrating the necessity of using a registration region that is wide enough to provide clinically practical information on mandibular asymmetry. **a** The mirrored (yellow) and original (gray) mandibular models were separated extensively and considerably when superimposed on the mandibular body mesial to bilateral canines. **b** The location and extent of asymmetry shown through superimposing on a wider region (i.e., the mandibular body mesial to bilateral second molars) seemed more rational to be used to make the diagnosis and guide the treatment planning for mandibular asymmetry

Selection of the registration region for mirrored and original mandibular models is crucial in evaluating the asymmetry, especially for mandibles with remarkable unilateral deformities, as in CFM. Two important considerations motivated the selection. First, the registration region had to be wide enough and selectively localize the asymmetry, confirming the diagnosis and ensuring that treatment plans would be feasible and efficient. If the registration region was too small, as was tested by superimposing on the mandibular body mesial to bilateral canines, the mirrored and original models would separate from each other extensively and considerably (Fig. 4). Such results failed to provide clinically practical information and diagnosis of asymmetry. Because the principle locus of the CFM deformity is the ramus [26, 27], this area was excluded from the registration region.

Second, the registration region should be symmetric in terms of height, starting from the alveolar border where the adjustability and treatment options are limited mainly through orthodontic tooth movements. In that event, the height discrepancy would be concentrated at the lower border of the mandibular body. From a clinical point of view, this method facilitates comparison of the effectiveness and efficiency among treatment options, including surgical resection, augmentation, or orthodontics, or a combination depending on the asymmetry indicated by superimposition of the mandibles. For example, a body height excess of 3 mm can be more efficiently corrected with surgical resection if there are no concerns about nerve proximity, while orthodontics is also effective but less efficient and stable. Ultimately, a registration region mesial to the second molars was selected, similar to the choice of Solem et al. (i.e., a region mesial to the first molars) [28]; however, the current work included an additional criterion for height symmetry of the registration region to support devising feasible and efficient treatment strategies in cases with this asymmetry.

Ramal malformation is a typical and distinguishing characteristic of CFM, and many studies have reported that the ramus on the affected side is shorter than on the contralateral side [12, 24, 26, 27, 36]. However, no consensus exists regarding the influence of CFM on the mandibular body, possibly because of difficulty in measuring this body size in 2D images (i.e., cephalograms, orthopantomograms) and small sample sizes (i.e., 4–6 patients) in the 3D studies analyzing the body length [25, 26]. A recent CT study showed shorter mandibular bodies on the affected side for 28 CFM patients, including children and adults [27]. This finding is consistent with the current results. The significantly shorter body and ramus on the affected compared with the contralateral side suggest that CFM influences both. Although the extent of the body length asymmetry was less than that of the ramal length asymmetry, the mean length difference in the mandibular body between bilateral sides was as high as 8.90 mm (Table 5). This level of difference would be clinically significant for facial asymmetry. Thus, in addition to the main mandibular ramal discrepancy, the body discrepancy should be addressed when planning treatment.

The association of the mandibular asymmetry and other mandibular characteristics with the most commonly used CFM classification system (i.e., Pruzansky–Kaban classification system) was explored, which was expected to reinforce its practical applicability in the diagnostic process. Using this system, mandibles in the severe group were smaller (i.e., shorter body and ramal lengths) on both the affected and contralateral sides and more retruded (i.e., smaller ANB and SNB angles) than in the mild group. This result is in agreement with those of previous studies conducted mainly with growing patients [17, 35, 36]. The SNB angle was smaller in the severe than in the mild group, although not significantly so. This lack of statistical insignificance might trace to the small numbers in this severe group. As for mandibular asymmetry, the severe group showed a significantly greater extent of body and ramus length asymmetry compared with the mild group. In contrast, the mandibular asymmetry score, a combined quantification of the size and shape asymmetry, was not significantly correlated with severity. The correlation between the asymmetry score and body length asymmetry was also weak, although significant. These results could be attributed to the wide shape diversity of CFM mandibles or to size asymmetry in the other two dimensions (e.g., ramal width, body height).

A broad variety of shape asymmetries in the mandibles of CFM was observed here. Greater shape asymmetry may manifest in CFM regardless of the severity of length asymmetry or mandibular deformity. Superimposition of mirrored and original mandibular models identified an outward displacement of the affected ramus relative to the contralateral ramus in 32 cases and an inward displacement and overlapping in 11 and 3 cases, respectively (Fig. 3). The outward displacement of the affected ramus was more prevalent in mandibles with type I and IIA deformities than in those with type IIB deformity. Among the 11 cases in which the affected ramus was displaced inside the mirrored contralateral ramus, three type IIB cases showed an outward bending toward the ipsilateral glenoid fossa in the upper part of the affected ramus (Fig. 3). This trend to outward displacement or bending of the affected ramus might help retain articulation of the temporomandibular joint complex. Consistently, previous studies analyzing mandibular growth in CFM have demonstrated lateral growth of the condyle on the affected side [28, 35].

One limitation of this study was the small sample sizes because of the low prevalence of CFM, especially for the severe group (i.e., types IIB and III). A larger number of patients could have enhanced the statistical power of our analyses. However, sample sizes of previously published CT or CBCT studies of the CFM mandible have all involved fewer than 30 patients, usually with mild CFM types. The other limitation was that the assessment of the size asymmetry in this study was focused on length. Mandibular size asymmetry covering the other two dimensions (e.g., body height, ramal width, volume) and positional asymmetry should be investigated to further improve understanding of the asymmetric pathology of CFM.

## Conclusions

For adults with unilateral CFM, the lengths of the mandibular body and ramus were significantly shorter on the affected side than on the contralateral side. An increased severity of mandibular deformity based on the Pruzansky–Kaban classification was associated with mandibles that were smaller, more retruded, and more asymmetric in length. On the other hand, the mandibular asymmetry score, which was a combined quantification of 3D size and shape asymmetry of the mandible, showed no correlation with the deformity severity and only a weak correlation with body length asymmetry. This result could be explained by the high variability in shape asymmetry among the mandibles. Despite this broad shape variability, an outward displacement of the affected ramus was observed in more than half of the cases. The Pruzansky–Kaban classification supports the diagnosis, but clinicians should be aware that high morphologic variability exists within each type and consider this factor in treatment planning.
